# Enterovirus D68 epidemic, UK, 2018, was caused by subclades B3 and D1, predominantly in children and adults, respectively, with both subclades exhibiting extensive genetic diversity

**DOI:** 10.1099/mgen.0.000825

**Published:** 2022-05-09

**Authors:** Hannah C. Howson-Wells, Theocharis Tsoleridis, Izzah Zainuddin, Alexander W. Tarr, William L. Irving, Jonathan K. Ball, Louise Berry, Gemma Clark, C. Patrick McClure

**Affiliations:** ^1^​ Clinical Microbiology, Nottingham University Hospitals NHS Trust, Nottingham, UK; ^2^​ School of Life Sciences, University of Nottingham, Nottingham, UK; ^3^​ Wolfson Centre for Global Virus Research, University of Nottingham, Nottingham, UK

**Keywords:** virology, enterovirus, enterovirus 68, human, EV-D68, enterovirus 68, molecular epidemiology, genotype, genetic epidemiology

## Abstract

Enterovirus D68 (EV-D68) has recently been identified in biennial epidemics coinciding with diagnoses of non-polio acute flaccid paralysis/myelitis (AFP/AFM). We investigated the prevalence, genetic relatedness and associated clinical features of EV-D68 in 193 EV-positive samples from 193 patients in late 2018, UK. EV-D68 was detected in 83 (58 %) of 143 confirmed EV-positive samples. Sequencing and phylogenetic analysis revealed extensive genetic diversity, split between subclades B3 (*n*=50) and D1 (*n*=33), suggesting epidemiologically unrelated infections. B3 predominated in children and younger adults, and D1 in older adults and the elderly (*P*=0.0009). Clinical presentation indicated causation or exacerbation of respiratory distress in 91.4 % of EV-D68-positive individuals, principally cough (75.3 %), shortness of breath (56.8 %), coryza (48.1 %), wheeze (46.9 %), supplemental oxygen required (46.9 %) and fever (38.9 %). Two cases of AFM were observed, one with EV-D68 detectable in the cerebrospinal fluid, but otherwise neurological symptoms were rarely reported (*n*=4). Both AFM cases and all additional instances of intensive care unit (ICU) admission (*n*=5) were seen in patients infected with EV-D68 subclade B3. However, due to the infrequency of severe infection in our cohort, statistical significance could not be assessed.

## Data Summary

The genetic data that support the findings of this study are openly available in GenBank at https://www.ncbi.nlm.nih.gov/genbank/, accession numbers MZ576283–MZ576352. Supporting data and protocols have been provided within the article and supplementary data files and additional raw data that further support the findings of this study are available from the corresponding author upon reasonable request.

**Impact Statement**

Enterovirus D68 (EV-D68) has been identified as a cause of biennial epidemics of respiratory disease around the world and a probable cause of acute flaccid myelitis/paralysis (AFM/AFP), a severe neurological complication similar to polio, another enteroviral disease. To better understand this emerging pathogen, we looked at both the viral genomic and human features of the most recent EV-D68 infection wave (2018) in patients cared for at a regional UK hospital. Our relatively large cohort showed that whilst this epidemic season was composed of two genomic subtypes (B3 and D1 in a 60 : 40 split), there was further variation within each. These data also suggested a considerable undiagnosed burden of transmission and infection both nationally and internationally. EV-D68 subtype B3 predominated in children and younger adults and D1 in older adults and the elderly. The vast majority of the 83 cases presented with respiratory illness, some severe and requiring intensive care. Neurological symptoms were rare (four patients), but included two confirmed AFM diagnoses. Future EV-D68 epidemic trajectory is uncertain due to coronavirus disease 2019 (COVID-19) pandemic intervention measures, but this study establishes a detailed genetic and clinical picture of EV-D68 disease at a single UK centre with which to compare post-pandemic EV-D68 epidemics.

## Introduction

The genus *Enterovirus* (EV) within the non-enveloped ssRNA family *Picornaviridae* includes seven species infecting humans: three rhinovirus species (*Rhinovirus A–C*) and four enterovirus species (*Enterovirus A–D*) [1]. These EV species are further divided into distinct genotypes, prefixed with A, B, C or D to indicate their species assignment. EV infections in humans are caused by in excess of 100 genotypes and manifest as a diverse array of symptoms from asymptomatic infection to respiratory, epidermal, gastrointestinal and neurological presentations [2]. Globally, EV infections represent a considerable burden of morbidity and mortality [2, 3], and they remain the leading cause of viral meningitis worldwide.

Since its recent re-emergence, enterovirus D68 (EV-D68) has been associated with biennial (2014, 2016 and 2018) seasonal epidemics of respiratory infections and, more rarely, acute flaccid myelitis (AFM) across Northern America [4–6], with subsequent identification of similar patterns globally [7–9]. There are limited studies looking at the true burden of EV-D68. Multiple contemporary strains of EV-D68 have been associated with neuropathology both *in vitro* [10] and in mouse models following dissemination to the central nervous system [11, 12], with others suggesting this is a universal EV-D68 phenotype including the archetypal 1962 Fermon strain [13]. Despite its neurotropic nature, EV-D68 is primarily an infection of the respiratory tract and, in contrast to many other enterovirus species, transmission is primarily by airborne routes and fomites [14, 15]. EV-D68 is infrequently found in faeces [16] and is not thought to spread by the faecal–oral route, as it is acid labile [2, 17]. There are four identified EV-D68 clades, designated A–D, with subclades B3 and D1 contemporaneously predominating in global sequence data [8, 18–22]. Evidence is emerging to suggest that the severity of EV-D68 infection is associated with the presence of other co-morbidities [23]. This large retrospective study also highlighted an upsurge in D1 cases in the adult population at a time when B3 and D1 were co-circulating.

Serological studies have retrospectively indicated an increasing population immunity against EV-D68 since at least 2006 [14, 24, 25], highlighting a growing importance and foothold in the roster of common childhood infections. The detection of enterovirus antibodies in the cerebrospinal fluid (CSF) of patients with an AFM diagnosis [26, 27] provides further evidence to link enterovirus infection and AFM, although EV-D68 RNA is rarely detected in CSF [7, 28–30].

Enterovirus infections are now routinely diagnosed by molecular methods and are further sub-typed by neutralization or sequence-based methods [2]. Sequencing of the VP1 gene, encoding one of four viral capsid proteins, is considered the current diagnostic typing gold standard [31], rather than the highly conserved 5′ untranslated (5′ UTR) genomic region, due to recombination events observed within this genomic region [32]. The recent re-emergence of genetically divergent EV-D68 strains (in relation to the prototype Fermon strain) [25, 26] may impair their detection by some nucleic acid-based commercial assays, as these are often designed exclusively with the historical Fermon strain. As such, commercial assays may have reduced analytical sensitivity due to possible primer mismatching [28].

This study retrospectively rescreened EV-positive samples from patients assessed at a regional UK hospital between September and December 2018 to determine the prevalence, genetic relatedness and clinical characteristics of EV-D68 infection. Two simple, novel EV-D68-specific RT-PCR assays were designed for both qualitative detection and sequencing of the VP1 gene. VP1 sequences were compared to the entire EV-D68 global dataset and clinical characteristics were audited to assess subclade phenotype.

## Methods

### Specimens

Original samples were identified to be EV-positive by routine diagnostic testing for EV RNA by Nottingham University Hospitals (NUH) Clinical Microbiology department, East Midlands, UK, between 2 September and 15 December 2018. This included total nucleic acid (TNA) extraction (bioMérieux NucliSENS easyMAG system) in a 50 µl elution volume and EV RNA detection by the AusDiagnostics Respiratory Viruses (16-well) (REF 20602) and/or Viral 8-well (ref 27093) assays for the *High*-Plex 24 system (REF 9150). The AusDiagnostics respiratory virus panel allows for non-specific detection of both rhinovirus (RV) and enterovirus (non-differentiated dual target: RV/EV), and specific detection of EV, in TNA. All TNA extracts were subsequently stored at −80 °C.

To prevent bias on analysis, TNA extracts were deduplicated to ensure that only one specimen per patient was investigated. If multiple TNA extracts were available from the same anatomical site, the earliest TNA extract was selected. All available residual TNA extracts of sufficient volume were included here (*n*=193) and derived from: throat swabs (*n*=117) and nasopharyngeal aspirates (*n*=41), swabs from other sites (*n*=19), CSF (*n*=7), sputum (*n*=4), faeces (*n*=2), bronchoalveolar lavage (*n*=2) and whole blood (*n*=1).

### RT-PCR, primer design and sequencing

cDNA was synthesized and amplified from TNA extracts as previously described [33]. Samples were targeted with a generic 5′ UTR based assay generating a ~480 bp product as previously described [33, 34], and two novel EV-D68 specific assays: EV-D68 assay primers were designed following alignment of all available complete EV-D68 genomes (*n*=348) published on GenBank by November 2018 (NCBI: txid42789). Two overlapping amplicons were generated to ensure full coverage of the EV-D68 VP1 gene: primers D68_1442F (CCCTGATAATACCTTGGATyAGTGG) combined with D68_2267R (GCTGARTTAATRCACATAAARGGTAT) and D68_2167F (GACACTnCArGCAATGTTyGT) with D68_2762R (CCTGTRTTRCAYTTRCAYCTTGCTAT) generated circa 820 and 600 bp products, respectively. PCR products of expected size were diluted 1 : 10 in molecular grade water and subjected to Sanger sequencing (Source BioScience, UK) with primers D68_2267R and D68_2167F, as appropriate. Sequence identity was assessed using National Center for Biotechnology Information (NCBI) Standard Nucleotide BLAST (blastn) and the Genome Detective online Enterovirus Genotyping Tool (version 2.9) [35]. Complete VP1 sequences were uploaded to GenBank under accession numbers MZ576283–MZ576352.

### Phylogenetic and statistical analyses

All publicly available complete VP1 sequences were retrieved from GenBank in June 2021 and aligned with the study sequences using Geneious Prime 2019.2.3. A phylogenetic tree was constructed from the alignment in IQTREE2 [36] by the maximum-likelihood method with the GTR+F+I model and 1000 SH-aLRT bootstraps. The distances between the identified subgroups within subclades D1 and B3 were calculated by summing the branch lengths to their common ancestral node based on the maximum-likelihood tree utilizing a GTR+F+I model of evolution. Clinical features were assessed in the two groups representing the B3 and D1 subclades. Comparison of continuous variables and categorical datasets were performed for the two groups using a binary logistic regression model, including the parameters age, gender, respiratory symptoms (cough, shortness of breath, coryza, wheeze, oxygen, sore throat, bronchiolitis, pneumonia), fever, suspected sepsis, underlying conditions, neurological symptoms and immunodeficiency. Analyses were performed with GraphPad Prism v.9.2.0.

## Results

### Enteroviral epidemiology in Nottingham, UK, autumn 2018

**Fig. 1.**
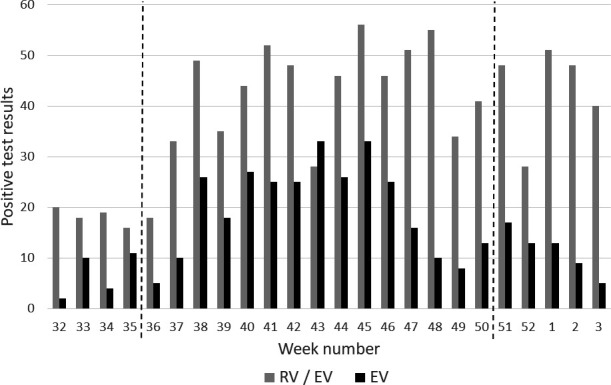
The number of respiratory specimens from which rhinovirus/enterovirus (RV/EV) and enterovirus (EV; species A–D) were detected between 2018 week 32 and 2019 week 3, as reported by NUH NHS Trust, Nottingham, UK. TNA from samples received from weeks 36 to 50 (marked by dashed lines) were subjected to further analysis.

Detection of RV/EV and EV in respiratory specimens increased from week 36 of 2018 (early September) and EV diagnoses remained elevated until week 45 (November, Fig. 1). RV/EV numbers remained high after the decline in EV, as had been seen for the corresponding period of 2017 (data not shown). Retrospective analysis of EV-positive TNA was performed on samples received for routine testing during this peak period of 2018, between weeks 36 and 50 inclusively. In total, 308 positive patients were identified, of which 193 residual TNA extracts from 193 patients had sufficient volume for further analysis.

To investigate the prevalence of EV-D68 in the cohort, two novel EV-D68-specific primer combinations, amplifying the VP1 capsid gene, were utilized. An additional novel pan-enterovirus specific control assay targeting the conserved 5′ UTR region was also employed [33].

Of these 193 EV clinical assay-positive extracts, our study assays confirmed EV in 143 (74.1 %, Table S1, available in the online version of this article). Of the confirmed EV-positive samples, EV-D68 RNA was detected in 58.0 % of cases (*n*=83), as determined by sequencing of products from either or both VP1 study assays, threefold greater in number than the next most prevalent EV type, CVA6 (Table S1). Clinical RT-PCR assay results of duplicate specimens obtained from EV-positive individuals that were excluded from this study are presented in Table S2.

The diagnostic results for the 50 (25.9 %) TNA extracts where EV RNA was not detected by our in-house EV assays were reviewed. Many presented with a low and potentially limiting EV value on initial screening (Fig. S1) . All other samples had a high ratio of combined RV/EV copy number to EV specific copy number (Fig. S2), indicating potential rhinoviral cross-reactivity, with some instances confirmed by sequencing (Table S1). No significant differences in copy numbers or ratios of RV/EV : EV were observed between either EV-D68 and non-EV-D68 or EV-D68 subclades (Figs S1 and S2).

### Enterovirus D68 epidemiology, 2018 weeks 36 to 50

**Fig. 2.**
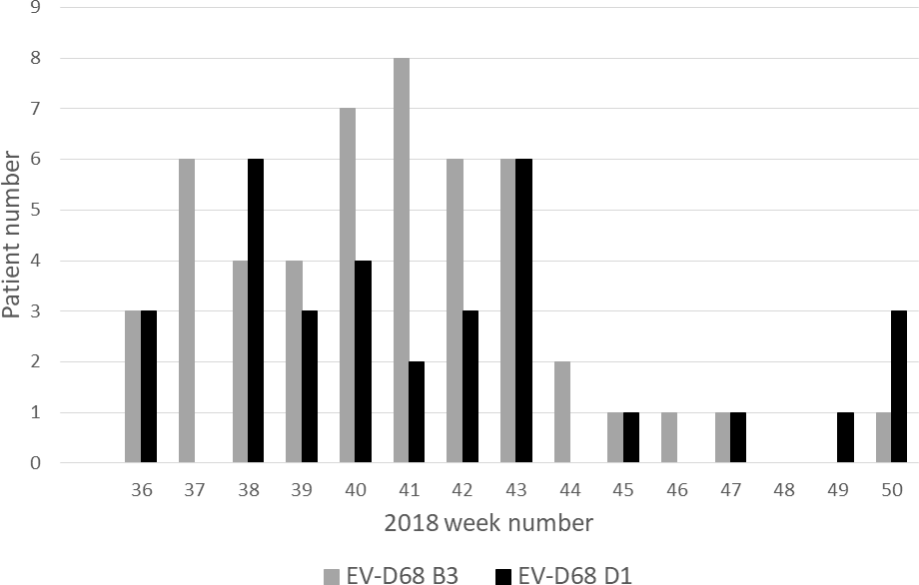
EV-D68 prevalence by individual patient and subclade, from weeks 36 to 50 of 2018 at NUH NHS Trust, Nottingham UK.

All amplicons were sequenced by Sanger methodology and typed using the Genome Detective online tool [35]. A full VP1 sequence was amplified and used for subsequent phylogenetic analysis in 70 of 83 (84.3 %) samples, but sequencing of a partial VP1 region in isolation still allowed for successful subtyping. Co-circulation of two EV-D68 subclades was observed, as reported concurrently elsewhere in Europe [19–21], with 50 subclade B3 and 33 subclade D1 positives identified (Fig. 2). EV-D68 B3 predominated in the peak epidemic period of weeks 40 and 41, whilst strains belonging to the less prevalent subclade D1 were present throughout the study period, with peaks seen in weeks 38 and 43 (Fig. 2).

**Fig. 3.**
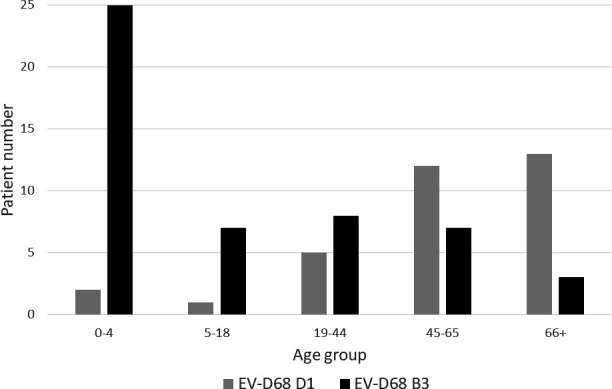
Age distribution of patients infected with EV-D68, stratified by subclade (**B3 and D1**).

Patients with EV-D68 B3 were significantly younger than those infected with subclade D1 (*P* value=0.0009, Table 1), with 64.0 % (*n*=32) of B3-positive patients ≤18 years of age (median 4.5 years, Table 1), and 78.1 % (*n*=25) of those patients under 5 years old (Fig. 3). In contrast, 91 % (*n*=30) of patients with EV-D68 subclade D1 were >18 years old (median 56 years, Table 1), with the majority (75.6%, *n*=25) of patients aged 45 years and over (Fig. 3).

**Table 1.** Clinical features of EV-D68 patients, Nottingham, UK, Autumn 2018

Characteristic	All EV-D68		EV-D68 B3		EV-D68 D1		Odds ratio range	*95 %* *C*	*P*-value	
**Age range**	23 days to 92 years		23 days to 92 years (median 4.5 years)		8 months to 89 years (median 56 years)		1.1	*1.037 to 1.136*	**0.0009**	***
	*n*	%	*n*	%	*n*	%				
**Female gender**	44	54.3	24	48	20	64.5	0.5	0.092 to 2.124	0.3101	ns
**Respiratory symptoms**	74	91.4	46	92	28	90.3	1.1	0.068 to 19.91	0.928	ns
***Cough***	61	75.3	37	74	24	77.4	4.1	0.502 to 37.37	0.1885	ns
***Shortness of breath***	46	56.8	29	58	17	54.8	2.2	0.422 to 13.05	0.3478	ns
***Coryza***	39	48.1	29	58	10	32.3	0.9	0.181 to 4.801	0.9457	ns
***Wheeze***	38	46.9	28	56	10	32.3	7.0	1.022 to 67.29	0.062	ns
***Oxygen required***	38	46.9	25	50	13	41.9	0.4	0.069 to 2.231	0.3143	ns
***Sore throat***	8	9.9	4	8	4	12.9	4.9	0.482 to 66.36	0.193	ns
***Bronchiolitis***	8	9.9	6	12	2	6.5	12.6	0.941 to 207.0	0.0554	ns
***Pneumonia***	7	8.6	1	2	6	19.4	1.7	0.098 to 67.89	0.7242	ns
**Fever**	31	38.3	19	38	12	38.7	0.6	0.124 to 2.928	0.5502	ns
**Suspected sepsis**	7	8.6	5	10	2	6.5	0.0	0.0005 to 1.100	0.0789	ns
**Co-morbidities**	46	56.8	23	46	23	74.2	3.1	0.627 to 19.11	0.1838	ns
**Neurological symptoms**	4	4.9	3	6	1	3.2	0.8	0.017 to 19.74	0.878	ns
**Immunocompromised**	14	17.3	9	18	5	16.1	0.1	0.004 to 0.4574	**0.0155**	*
AFM/ICU admission	2*/ 5	8.6	2*/5	14	0	0	na	na	na	na
**Total**	**81**	**100**	**50**	**61.7**	**31**	**38.3**				

Clinical parameters were determined from existing clinical records on each patient. Data with continuous variables, and categorical variables (including comorbidities, presence of fever, presence of neurological symptoms and previous evidence of immunodeficiency) were included in a binary logistic regression model. Subcategories of respiratory symptoms are italicised.

*One AFM patient was also admitted to ICU.

AFM, acute flaccid myelitis; CI, confidence interval; ICU, intensive care unit; na, not applicable; ns, non-significant.

### Clinical features of EV-D68-positive patients

Detailed clinical information was available for 81 of 83 EV-D68-positive individuals and analysis of a range of these features associated with either EV-D68 subclade D1 or B3 was performed using binary logistic regression (Table 1). This regression model indicated that in addition to age, immunocompromisation was also significantly associated with B3 infection. Overall, the model correctly predicted 85.19 % of cases. Respiratory symptoms were present for 91.4 % of patients, most commonly a cough (75.3%), shortness of breath (56.8%), coryza (48.1%), wheeze and a requirement for oxygen (both 46.9%). Fever was also frequently reported (38.3%), as were co-morbidities (56.8%). Neurological symptoms were only reported in four cases (4.9%), including two cases of AFM, one in a 22-year-old adult and the other in a 1-year-old child. In both cases EV-D68 subclade B3 was detected; in the CSF of the adult and, unusually, in the faeces of the paediatric AFM case. No other CSF or faecal samples in the study period were found to be EV-D68-positive. The adult AFM patient required intensive care, as did five other patients – a 20-year-old adult and four children aged 0–9 years. All five patients were infected with EV-D68 subclade B3.

### Genetic epidemiology of enterovirus D68 in Nottingham, UK, between September and December 2018

**Fig. 4.**
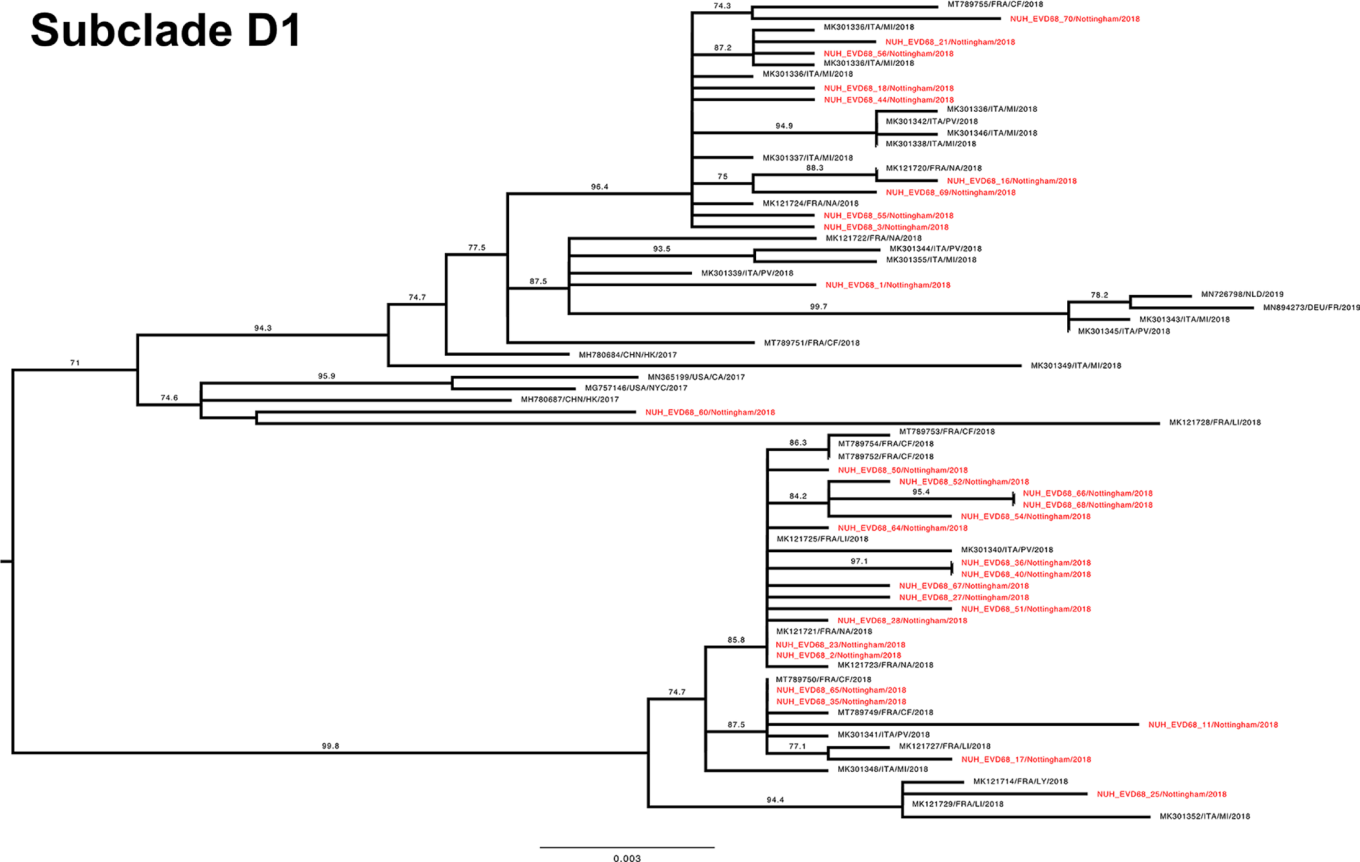
Phylogenetic relationship by maximum-likelihood method of Nottingham, UK, 2018 complete EV-D68 VP1 sequences (927–930 bp) designated subclade D1 (coloured in red) with all closely related publicly available genomes retrieved from GenBank in June 2021 (identified by accession number/country (with optional region)/year). The phylogeny depicted is a subtree of a complete tree with entire study and global sequence dataset presented in the appendix (Fig. S1). Numbers above individual branches indicate SH-aLRT bootstrap support, with values <70 not shown. Branch lengths are drawn to a scale of nucleotide substitutions per site, with scale indicated.

**Fig. 5.**
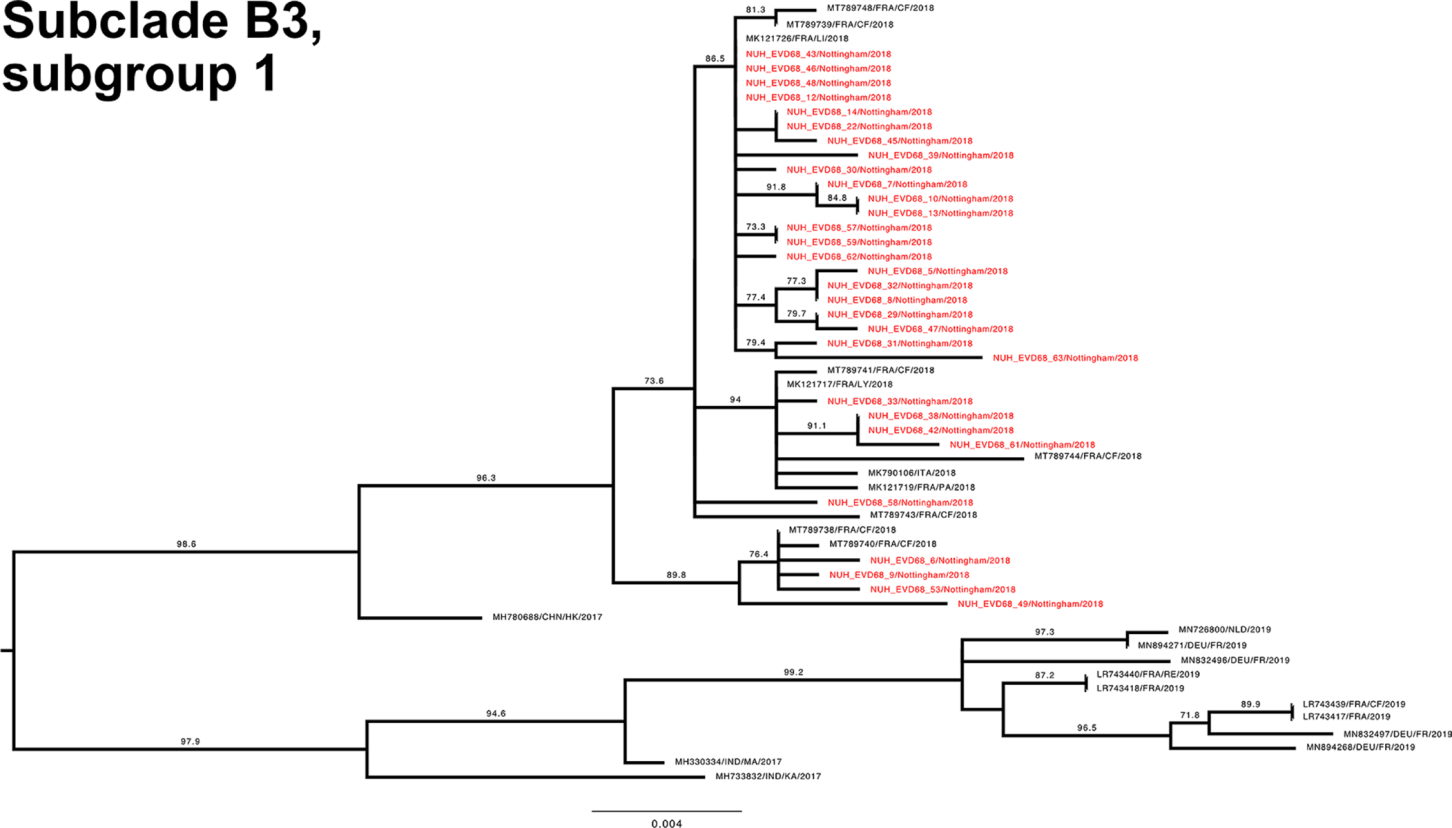
Phylogenetic relationship by maximum-likelihood method of Nottingham, UK, 2018 complete EV-D68 VP1 sequences (927–930 bp) designated subclade B3, subgroup 1 (coloured in red) with all closely related publicly available genomes retrieved from GenBank in June 2021 (identified by accession number/country (with optional region)/year). The phylogeny depicted is a subtree of a complete tree with entire study and global sequence dataset presented in the appendix (Fig. S1). Numbers above individual branches indicate SH-aLRT bootstrap support, with values <70 not shown. Branch lengths are drawn to a scale of nucleotide substitutions per site, with scale indicated.

**Fig. 6.**
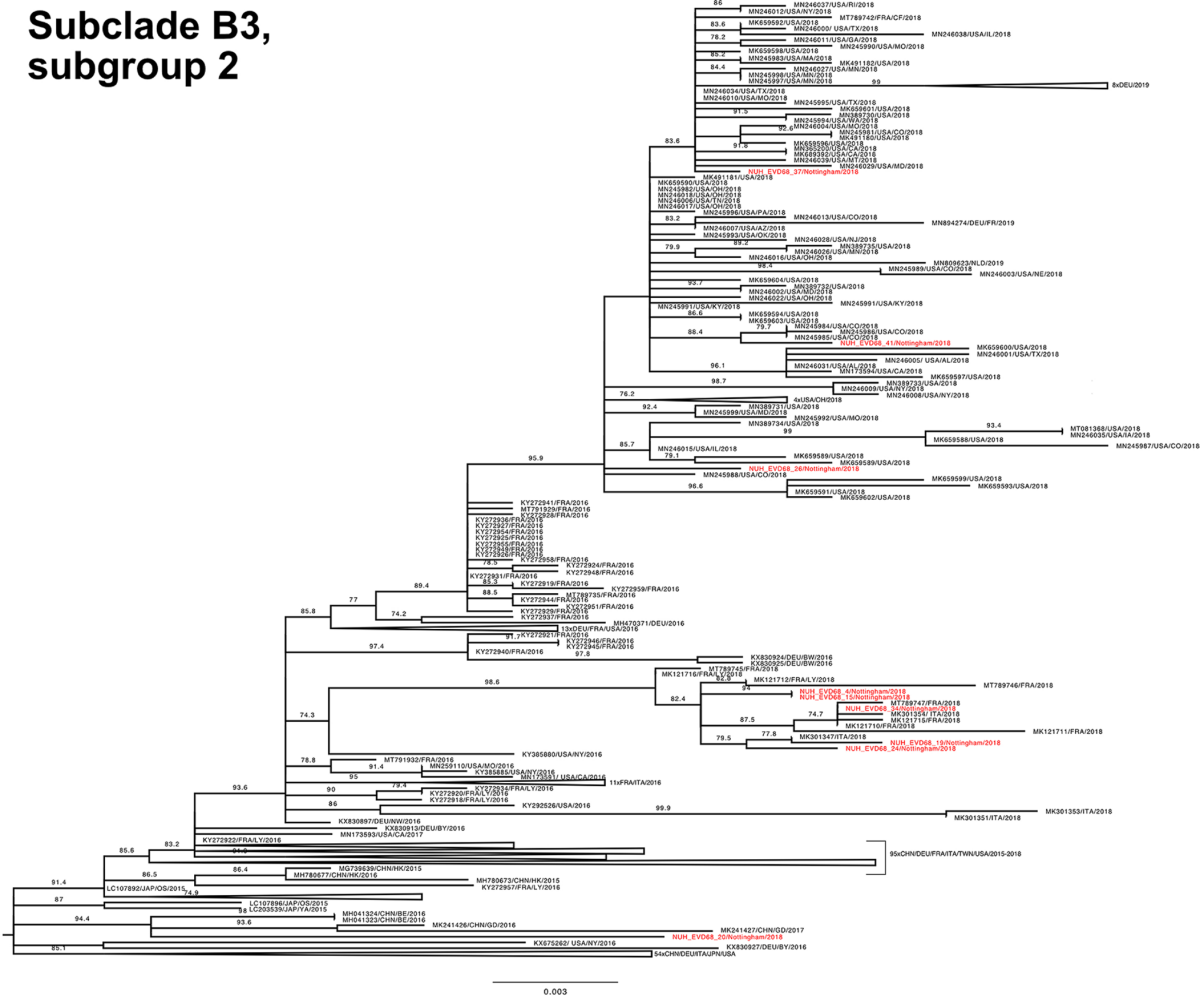
Phylogenetic relationship by maximum-likelihood method of Nottingham, UK, 2018 complete EV-D68 VP1 sequences (927–930 bp) designated subclade B3, subgroup 2 (coloured in red) with all closely related publicly available genomes retrieved from GenBank in June 2021 (identified by accession number/country (with optional region)/year). The phylogeny depicted is a subtree of a complete tree with entire study and global sequence dataset presented in the appendix (Fig. S1), with well supported branches containing no study sequences collapsed to improve clarity. Numbers above individual branches indicate SH-aLRT bootstrap support, with values <70 not shown. Branch lengths are drawn to a scale of nucleotide substitutions per site, with scale indicated.

To investigate the genetic relationship of the study EV-D68 strains, a phylogenetic tree with our 70 complete VP1 sequences and the entire available global dataset of circa 1500 sequences (Fig. S3) was constructed. Both subclade D1 (Fig. 4) and B3 (Figs 5 and 6) VP1 sequences were split across distinct phylogenetic subgroups within the total dataset.

The VP1 subtree harbouring all the D1 sequences from this study (Fig. 4) was most closely out-grouped, yet distinct from, both prior-2016 isolates from East Asia (e.g. MH780679 and KY358058), a contemporary 2018 French sample (MK121713 [20]) and later-2019 isolates from Europe (e.g. LR743441 [18]). The subtree containing all 30 full-length VP1 D1 sequences from this study and 39 database references could be subdivided into two further phylogenetic groups, separated by a genetic distance of 0.013. The first (Fig. 4, lower half) contained exclusively contemporary European isolates from investigations in France and Italy [19, 20], whereas the other (Fig. 4, upper half) additionally contained a well-supported subgroup with four 2017 sequences from the USA and Hong Kong, a 2018 French isolate (MK121728 [20]) and a single Nottingham sequence. Interestingly, this latter patient (NUH_EVD68_60) was the only one in the cohort to report recent travel history to East Asia. Our study sequences frequently shared a closer genetic relationship with contemporary isolates from continental Europe than with each other. For example, samples NUH_EVD68_21, 23 and 25 were all sampled within 24 h of each other, yet appeared in discrete, well-supported regions of the tree with a closer genetic identity to various European isolates than each other (Fig. 4).

The Nottingham EV-D68 B3 sequences (depicted across Figs 5 and 6 for improved clarity, but only separated by a basal genetic distance of 0.003) presented a similar picture of co-mingling with contemporary European isolates, with for example NUH_EVD68_34 residing in a well-supported cluster of EV-D68 B3 sequences with three French strains and one Italian strain (Fig. 6). By contrast, the chronologically adjacent UK sequence NUH_EVD68_33 sampled on the previous day appeared in an entirely different but equally well-supported subclade of B3 (Fig. 5).

Recent travel history was also noted for other patients in both clades (NUH_EVD68_4, 7, 20 and 27, Figs 4–6). Of note, NUH_EVD68_20 (Fig. 6), with recent Spanish travel history, presented in a distinct supported phylogenetic group with four Chinese sequences from 2016 and 2017 [37]. The other cases, with travel histories to the USA, Denmark and Spain, did not present any distinctive sub-groupings with reference sequences. However, NUH_EVD68_4 (Fig. 6), also with recent Spanish travel history, presented an identical VP1 sequence to NUH_EVD68_15 received 13 days later. Both these patients were 5 years old and shared the same hometown and outer UK postcode. There were eight other instances of identical pairs or clusters of study sequences, but clinical details did not suggest a direct familial or geographical association. Most were relatively close samples in terms of time, but NUH_EVD68_35 and 65 were received 2 months apart in different but adjacent UK outer postcodes.

Taken together, these observations would suggest both Europe-wide and local circulation of multiple strains of both B3 and D1 subclades underlying the autumn 2018 EV-D68 epidemic.

## Discussion

Enteroviruses can cause a wide range of severe clinical manifestations, notably in young children, and are a leading cause of meningitis globally [2]. Continued surveillance of enteroviruses within the UK, despite its polio-free status, is still highly relevant, notably highlighted by the recent resurgence and recurrent epidemic seasonality of EV-D68 with associated AFM globally and nationally in the UK [38–42].

Growing recognition of AFM and enteroviral association in autumn 2018, particularly in the UK [42], prompted us to further investigate enterovirus-positive samples using novel in-house RT-PCR assays and Sanger sequencing. Whilst this approach is well established and has more recently been over-shadowed by whole-genome sequencing using deep sequencing methods, it provides a flexible, inexpensive and fast route to enteroviral typing and epidemiology in a clinical setting.

We found a significant burden of EV-D68 infection in our 2018 study period between September and December, coinciding with an overall increase in RV/EV positivity, responsible for 58.0 % of 143 confirmed enteroviral positives, more than 10-fold higher than its overall recent EU prevalence of 4.53 % [2]. It is difficult to determine the context of this spike in EV-D68 positivity locally, relative to both the rest of 2018 and other years, without routine typing of all enterovirus-positive respiratory samples in addition to the more frequently assessed neurological symptom-associated enteroviral infections [2, 28, 33, 43]. EV-D68 is an endemic infection that is maintained in the population and is still detected in Europe outside of the currently expected biennial peak years [18], but resources were not available to routinely type respiratory samples outside of our focused study period.

We targeted the highly variable and type-indicative enteroviral VP1 region [28] with novel primers specific to EV-D68 to not only discriminate EV-D68 infection (no cross-reactivity was observed with other enteroviruses), but also determine the relative contribution by circulating clades. To the best of our knowledge, this allows us to present the largest fully typed EV-D68 cohort from a single centre and epidemic season. Whilst both clades were present throughout our sampling period, an approximate 60 : 40 predominance of subclade B3 relative to D1 was observed. This was the reverse of the clade prevalence seen in other European studies of the 2018 EV-D68 season in France [20] and Italy [19], but in agreement with other investigations of previous seasons in France [21], Italy [22] and more generally in Europe post-2018 [18].

Universal typing and relatively large cohort size enabled us to investigate EV-D68 subclade association with clinical characteristics with more statistical power than most other studies to date. Here we found a highly significant association between age and clade type, with subclade B3 predominantly affecting children and adolescents, whilst conversely D1 was seen in older adults and the elderly. This finding is in agreement with previous observations [8, 20, 22], but only one other study of sufficient size and scope to assign statistical significance [44].

EV-D68 seroprevelance in adults is almost universal, with primary infection seen frequently in the first 2 years of life [24, 25]. The predominance of the novel D1 subclade in hospitalized older adults is potentially multifactorial and may indicate an evolutionary adaptation of EV-D68 to evade a pre-existing immune response and facilitate repeat infection. Recent and rapid evolution of antigenic regions of the outer viral capsid corresponding to neutralizing epitopes has been suggested to be implicated [8, 22, 44]. Such adaptation may conversely present a general fitness or virulence cost, which could potentially explain the lower D1 detection in younger individuals, compared to B3, at least in our cohort with clinically significant disease, but such a hypothesis requires further investigation. Lineage-specific protection determined by a primary infection influencing virus-type age distribution has been described for several other viral infections, notably influenza A and B [45, 46].

Despite the significant differences in age profile for each subclade and our cohort size, no other significant difference was seen in clinical presentations recorded, in agreement with others [21]. The general clinical picture of symptoms indicative of both upper and lower respiratory tract infections was consistent with other studies [19, 20, 22, 30, 47]. Although one observation of this study was that all severe cases, including those with neurological involvement and an AFM diagnosis, presented with subclade B3, the infrequency of these severe presentations within our dataset inhibited robust statistical analysis from being performed and all cases were in younger individuals shown to be more likely to be infected with subclade B3. Indeed AFM is not restricted to the B3 subclade [4, 48, 49] and, in concordance with our data, is perhaps more likely associated with the predominant local strain infecting a naïve population and overall infection rates. Perhaps more notable in both of the AFM cases was the infrequently reported detection of viral genome in CSF and faeces; with EV-D68 being a predominantly respiratory pathogen and thus typically detected by sampling the respiratory tract [2, 50]. This finding supports a role for viral replication disseminated from the respiratory tract in EV-D68-associated AFM, although EV-D68 detection in sewage in the UK [51] and elsewhere [52] suggests faecal shedding may not be an unusual feature of infection in general.

We observed considerable genetic diversity within the VP1 regions of both the B3 and D1 subclades within our single epidemic season analysis, consistent and co-mingling with other contemporary European cohorts [19, 20]. In conjunction with the post-summer spike in case incidence, this is suggestive of a high level of community infection seeded by many regional and international introductions, as described in more detail elsewhere [44]. Our complete VP1 sequencing, in conjunction with a detailed clinical audit, provides greater granularity than many other studies. Notably, an individual with immediate East Asian travel history presented an EV-D68 VP1 sequence distinct from any other European isolates recorded to date, suggesting direct intercontinental importation. Other cases with recent travel history in Europe, combined with well-supported genetically related sequences from both Europe and the immediate local neighbourhood, are anecdotally suggestive of acquisition on holiday and onward transmission thereafter. Another case with Spanish travel history clustered exclusively with sequences from previous years in PR China, suggestive of considerable yet undetected intercontinental and international transmission chains occurring.

However, with minimal global surveillance, onward transmission from such introductions is difficult to ascertain with certainty in the short term, with prevailing introductions only apparent in subsequent epidemic periods. Yet despite this lack of resolution, EV-D68 VP1 sequencing assured local infection control teams in retrospect that individuals sharing hospital wards did not harbour genetically identical viruses and thus nosocomial transmission was unlikely and the infection prevention measures in place were effective.

We acknowledge several limitations in the work presented, principally the relatively short single time period assessed. Continuous and routine typing of all candidate EV-positive specimens received from an earlier outset would have provided a more complete picture and understanding of the evolving local EV-D68 burden. We thus could not assign a start and end point to our EV-D68 epidemic season. Recent initiatives such as the European non-polio enterovirus network [28, 38, 43] to which NUH NHS Trust Clinical Microbiology has contributed previously, will undoubtedly assist in increasing such surveillance. Working retrospectively with available diagnostic surplus further reduced both the sampling in the selected time period and also generally meant only single patient samples were available; thus for example general faecal shedding from patients with respiratory symptoms could not be investigated. We may also have overlooked some low-titre EV-D68-positive patients by not rescreening routine diagnostic assay-negative samples. Sub-optimal performance in diagnosing EV-D68 and other EVs by our chosen routine diagnostic assay and others has indeed been observed [43].

In summary, EV-D68 contributed significantly to the burden of enteroviral respiratory disease treated at our regional UK hospital between September and December 2018. Underlying this period of high prevalence were genetically distinct strains of EV-D68 subclades B3 and D1, which differed in their infected hosts’ age group, but no statistical significance in clinical presentation could be elucidated due to the low numbers for intensive care unit (ICU) admission and AFM cases in our cohort. Despite considerable contemporary concern and focus on the severe associated outcomes of EV-D68 infection, ultimately confirmed AFM cases were rare even in this largely hospitalized cohort. Nevertheless, the apparently increasing burden of EV-D68 and its diverse, rapidly evolving genome demand continued heightened surveillance.

## Supplementary Data

Supplementary material 1
