# Supplementary material for: Enterovirus D68 epidemic, UK, 2018, was caused by subclades B3 and D1, predominantly in children and adults, respectively, with both subclades exhibiting extensive genetic diversity
Source: Microb Genom. 2022 May 9;8(5):mgen000825. doi: 10.1099/mgen.0.000825 (PMC9465064; doi:10.1099/mgen.0.000825)
Supplement: Supplementary material 1 [file mgen-8-825-s001.pdf]

Supplementary material

Supplementary Table 1: *Enterovirus* species determined by in house study RT-PCR assays and Sanger sequencing.

***Enterovirus* species by sequencing result#**	**Number**	**Specimen site, listed by abundance (*n* shown in parenthesis)**
EV-D68	84*	Throat swab (62), NPA (20), CSF (1), sputum (1)
Enterovirus B, undetermined type	29	Throat swab (16), CSF (5), NPA (4), other swab (3), faeces (1)
CVA6	28	Other swab (13), Throat swab (11), NPA (2), faeces (1), whole blood (1)
CVA4	1	Throat swab (1)
Enterovirus C, undetermined type	1	Throat swab (1)
Rhinovirus C	3	NPA (2), Throat swab (1)
Product of expected size, but unreadable sequence	5	Throat swab (4), CSF (1)
No PCR product of expected size observed	42	Throat swab (21), NPA (13), other swab (3), sputum (3), BAL (2)

*#EV-D68 was confirmed by sequencing of the VP1 region; all other species preliminarily typed by sequencing of c.400bp of 5' UTR. One sample was positive for both EV-D68 and CVA6, but is included here as EV-D68. * One sample was EV-D68 positive by the 5' UTR assay, but not by either study EV-D68 specific VP1 assay. NPA: nasopharyngeal aspirate, CSF: Cerebrospinal fluid, BAL: Bronchoalveolar lavage.*

Supplementary Table 2: Study samples collected from EV-D68 cases alongside alternative samples (in italics) that were removed during the deduplication process.

**Study ID / additional sample**	**Age**	**Sex**	**Collection date**	**Sample Type**	**Clinical Assay RT-PCR Copy no. / 10µl**
NUH_EVD68_43	73	F	10/10/2018	Throat swab	RV/EV: 77444 EV:70719
NUH_EVD68_43/2	*73*	*F*	*11/10/2018*	*Throat swab*	*RV/EV: 21910 EV: 17445*
NUH_EVD68_45	42	M	13/10/2018	Throat swab	EV: 1510
NUH_EVD68_45/2	*42*	*M*	*13/10/2018*	*Throat swab*	*EV: 777*
NUH_EVD68_45/3	*42*	*M*	*14/10/2018*	*Throat swab*	*EV: 12*
NUH_EVD68_58	22	M	22/10/2018	Cerebrospinal Fluid	EV: 18
NUH_EVD68_58/2	*22*	*M*	*25/10/2018*	*Throat swab*	*RV/EV: 494 EV:2*
NUH_EVD68_58/3	*22*	*M*	*25/10/2018*	*Bronchoalveolar Lavage*	*RV/EV: 4069 EV:44*
NUH_EVD68_58/4	*22*	*M*	*27/10/2018*	*Swab (site not given)*	*RV/EV: Not detected*
NUH_EVD68_61	1	F	09/11/2018	Nasopharyngeal Aspirate	RV/EV: 773 EV: 599
NUH_EVD68_61/2	*1*	*F*	*14/11/2018*	*Faeces*	*EV: 18*
NUH_EVD68_61/3	*1*	*F*	*16/11/2018*	*Nasopharyngeal Aspirate*	*RV/EV: 27569 EV: 23454*
NUH_EVD68_61/4	*1*	*F*	*16/11/2018*	*Cerebrospinal Fluid*	*RV/EV: negative*

NB Only additional samples tested within one week of index sample have been presented to avoid confusion with additional investigations and/or infections potentially unrelated to this episode.

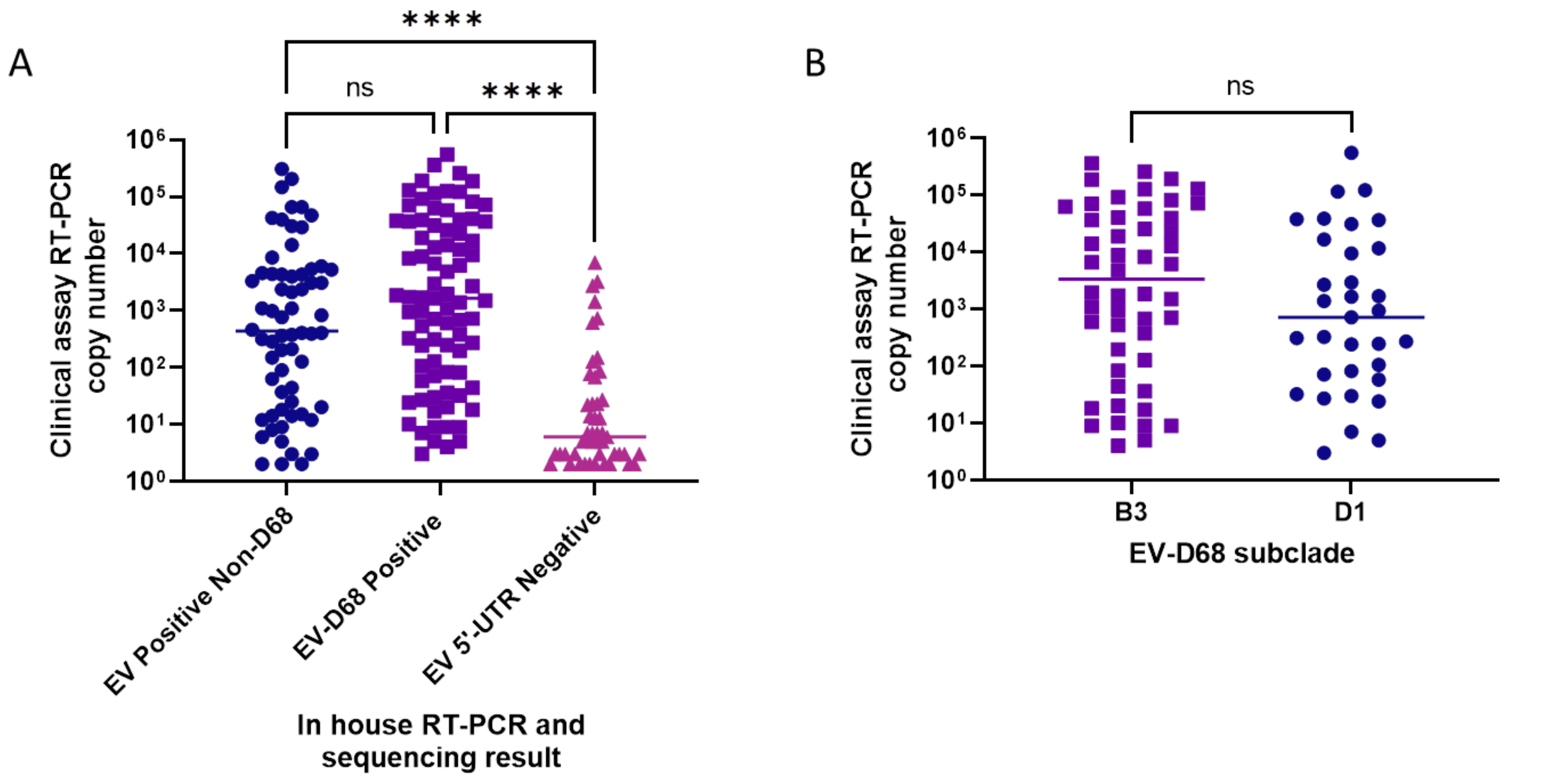

Supplementary Figure 1. EV RNA copy number per 10µl nucleic acid extract as reported by the clinical AusDiagnostics RT-PCR assay and categorised by in house RT-PCR assay and sequencing result (panel A) or EV-D68 subclade (panel B). Statistical significance of median difference was analysed by (A) Kruskal-Wallis and (B) Mann-Whitney tests, with indicated values either ****: p<0.0001, or ns: non-significant.

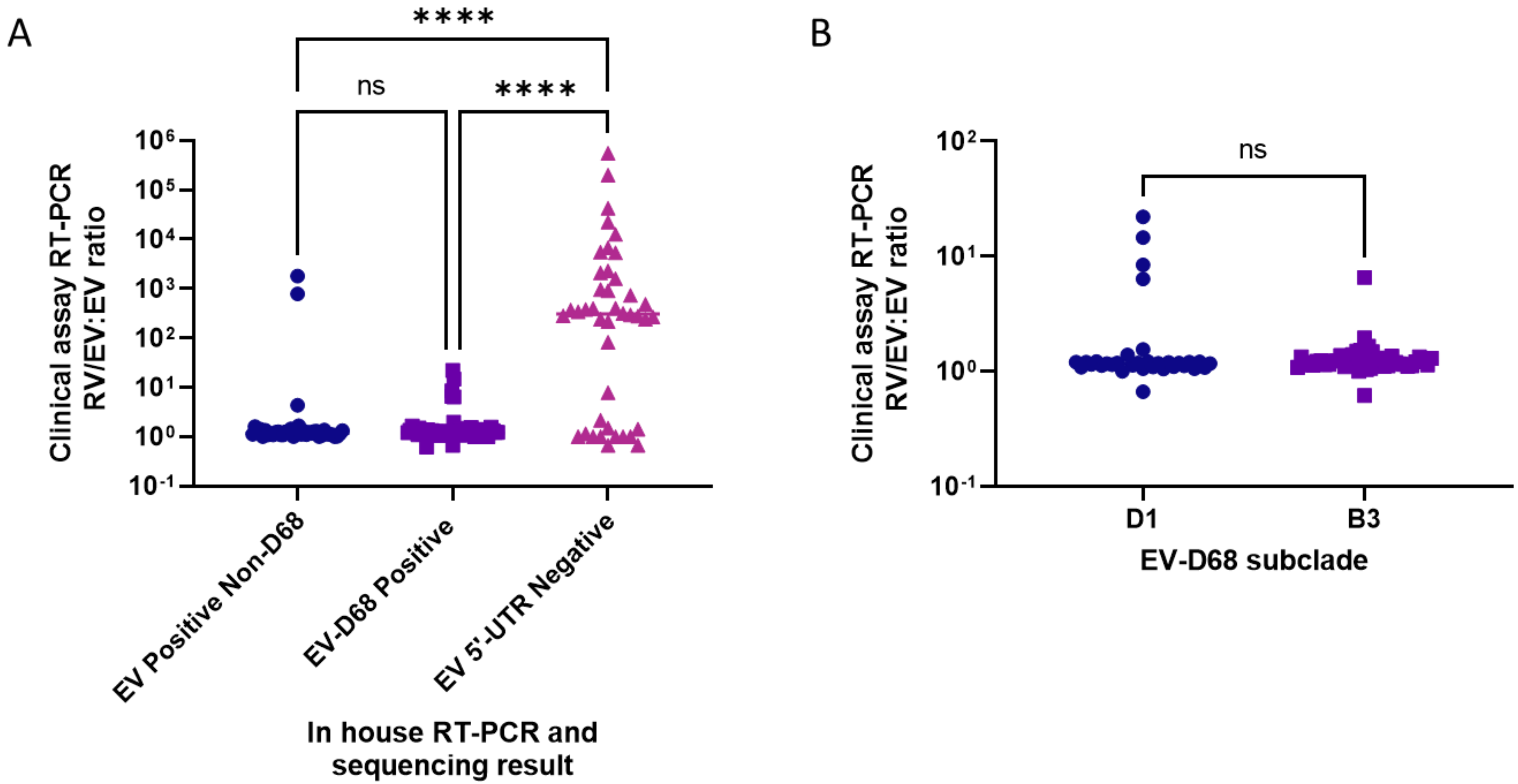

Supplementary Figure 2. Ratio of RV/EV:EV RNA copy number per 10µl nucleic acid extract as reported by the clinical AusDiagnostics RT-PCR assay and categorised by in house RT-PCR assay and sequencing result (panel A) or EV-D68 subclade (panel B). Statistical significance of median difference was analysed by (A) Kruskal-Wallis and (B) Mann-Whitney tests, with indicated values either ****: p<0.0001, or ns: non-significant.

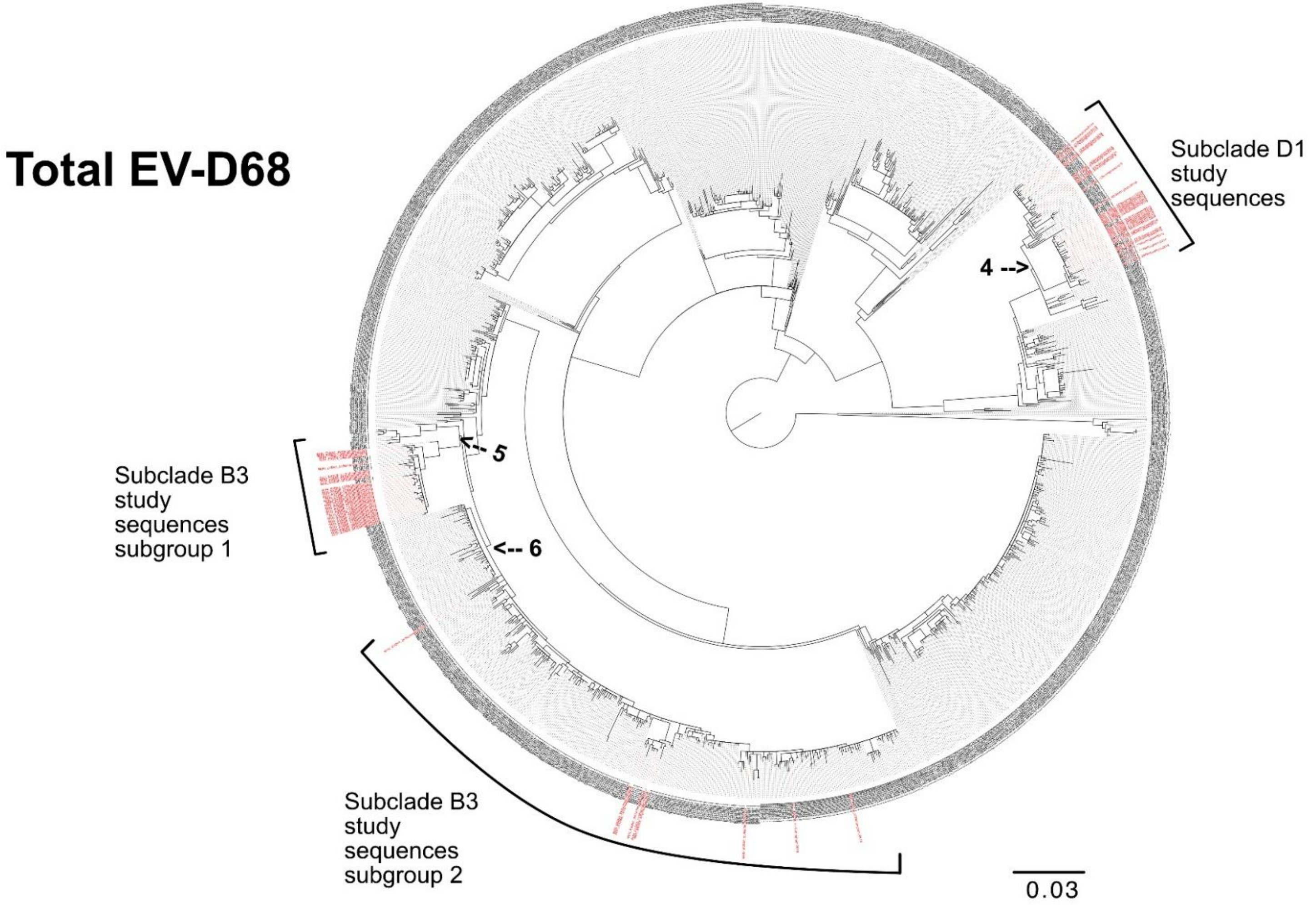

Supplementary Figure 3. Phylogenetic relationship by maximum likelihood method of Nottingham, UK, 2018 complete EV-D68 VP1 sequences (927-930 bp, coloured in red) with all publicly available genomes retrieved from GenBank in June 2021 (identified by accession number). Positions of subtrees presented in main document as Figures 4, 5 & 6 in main text are annotated 4, 5 & 6 respectively. SH-aLRT bootstrap support values have been omitted for clarity. Branch lengths are drawn to a scale of nucleotide substitutions per site, with scale indicated.
